# Impact of seven years of mass drug administration and recrudescence of *Schistosoma haematobium* infections after one year of treatment gap in Zanzibar: Repeated cross-sectional studies

**DOI:** 10.1371/journal.pntd.0009127

**Published:** 2021-02-12

**Authors:** Lydia Trippler, Shaali Makame Ame, Jan Hattendorf, Saleh Juma, Salum Abubakar, Said Mohammed Ali, Fatma Kabole, David Rollinson, Stefanie Knopp

**Affiliations:** 1 Swiss Tropical and Public Health Institute, Basel, Switzerland; 2 University of Basel, Basel, Switzerland; 3 Public Health Laboratory—Ivo de Carneri, Pemba, United Republic of Tanzania; 4 Neglected Diseases Program, Ministry of Health, Zanzibar, United Republic of Tanzania; 5 Department of Life Sciences, Natural History Museum, London, United Kingdom; World Health Organization, SWITZERLAND

## Abstract

**Background:**

Considerable progress towards the elimination of urogenital schistosomiasis was made by the Zanzibar Elimination of Schistosomiasis Transmission project from 2012 till 2016, when biannual praziquantel mass drug administration (MDA) alone or with additional snail control or behaviour change interventions were implemented. Annual MDA was continued in 2017 and 2018, but not in 2019, imposing a 16-month treatment gap. We monitored the *Schistosoma haematobium* prevalence from 2012 till 2020 and assessed recrudescence patterns with focus on 2020.

**Methodology:**

Repeated cross-sectional surveys were conducted from 2011/12 till 2020 in 90 communities and 90 schools in Zanzibar. Annually, around 4,500 adults and up to 20,000 schoolchildren were surveyed. The *S*. *haematobium* prevalence was detected by urine filtration and reagent strips. In 2020, risk factors for infection were investigated using generalized estimated equation models.

**Principal findings:**

In adults, the apparent *S*. *haematobium* prevalence was 3.9% in 2011 and 0.4% in 2020. In schoolchildren, the prevalence decreased from 6.6% in 2012 to 1.2% in 2019 with vicissitudes over the years. Prominent recrudescence of infection from 2.8% in 2019 to 9.1% (+225%) in 2020 was observed in 29 schools with historically moderate prevalences (≥10%). Compared with 2019, reinfection in 2020 was particularly striking in boys aged 9–16 years. Being male was a risk factor for infection in 2020 (adults: odds ratio (OR): 6.24, 95% confidence interval (95% CI): 1.96–19.60; schoolchildren: OR: 2.06, 95% CI: 1.52–2.78). Living near to a natural freshwater body significantly increased the odds of infection in adults (OR: 2.90, CI: 1.12–7.54).

**Conclusions/Significance:**

After 11 rounds of MDA over 7 years and a 16-month treatment gap, the urogenital schistosomiasis prevalence considerably rebounded in hotspot areas. Future elimination efforts in Zanzibar should focus on re-intensifying MDA plus additional interventions in hotspot areas. In low-prevalence areas, the strategy might be adapted from MDA to targeted surveillance-response.

## Author summary

Schistosomiasis is a neglected tropical disease caused by parasitic blood flukes of the genus *Schistosoma*. On the Zanzibar islands, United Republic of Tanzania, interventions to eliminate urogenital schistosomiasis commenced in 2012. From 2012 to 2016, the population was treated biannually with praziquantel and, additionally, some areas received mollusciciding against the intermediate host snail, or educational measures for behavior change. Mass drug administration (MDA) with praziquantel was continued annually in 2017 and 2018, but not in 2019. As a result of the interventions, the overall *S*. *haematobium* prevalence was reduced to 0.4% in adults and 3.4% in schoolchildren in 2020. However, in some areas, the MDA gap in 2019 resulted in a considerable rebound of infections. The recrudescence in 2020 was particularly striking for boys aged 9–16 years. In general, in 2020, male participants had higher odds of infection than females. Adults living near to a natural freshwater body also showed an increased risk of *S*. *haematobium* infection. Future elimination efforts in Zanzibar should focus on re-intensifying elimination interventions, including MDA, snail control and behavior change in hotspot areas. In low-prevalence areas, the strategy might be adapted from MDA to targeted interventions, such as surveillance-response.

## Introduction

The neglected tropical disease (NTD) schistosomiasis is endemic in 78 countries worldwide and, according to estimates from 2017, it is responsible for about 1.4 million disability-adjusted life years annually [1,2]. In their new road map for NTDs 2021–2030, the World Health Organization (WHO) highlights the global elimination of schistosomiasis as a public health problem as target for 2030 [3]. Elimination as public health problem is therein defined as <1% proportion of heavy intensity infections [3]. According to mathematical modelling as well as multi-country and multi-year studies, elimination of schistosomiasis as public health problem can be achieved relatively quickly [4–6]. Once countries or areas have achieved this goal, they likely wish to move further down the elimination road, aiming for the interruption of schistosomiasis transmission, defined as zero incidence of infection.

Clearly, as long as interruption of transmission has not been achieved, recrudescence of transmission and disease remains a threat. Recrudescence of transmission can easily occur due to the asexual reproduction of the parasite in its intermediate host snail and the release of thousands of cercariae into natural freshwater, which are then able to infect the next definitive human host [7,8]. Hence, as long as some, even very few people remain infected with schistosomes, the parasite’s lifecycle can be perpetuated and the risk of recrudescence remains [9]. Also after the transmission of the parasite was successfully interrupted in an area or country, transmission can be reintroduced as long as the intermediate host snails thrive and *Schistosoma* eggs are released into the snails’ freshwater habitat. To avoid recrudescence in areas where interruption of transmission has not yet been achieved, control and elimination efforts need to be maintained. To prevent reintroduction of schistosomiasis in areas considered post-elimination settings, a sensitive detection and timely treatment of cases through effective surveillance-response mechanisms is necessary [3]. Regarding the clonal reproduction of the parasite in its intermediate host snail, the detection of the parasite in the snail by xenomonitoring will constitute an important part of surveillance activities [7,8].

Interventions to control the morbidity caused by urogenital schistosomiasis in Zanzibar started in the 1980`s when *S*. *haematobium* was highly prevalent on the islands Pemba and Unguja [10–13]. Control interventions were enforced by large-scale mass drug administration (MDA) campaigns in schools implemented in the early 2000s [14–16]. These interventions paved the way for one of the first projects to eliminate urogenital schistosomiasis from an area in sub-Sahara Africa: the Zanzibar Elimination of Schistosomiasis Transmission (ZEST) project, implemented in Pemba and Unguja from 2011 till 2017 [17,18]. Over the course of ZEST, considerable progress towards the elimination of urogenital schistosomiasis was made [6,19]. During the ZEST project, MDA with praziquantel was implemented biannually in schools and whole communities across the islands Pemba and Unguja. In addition to biannual MDA, snail control and behavioural change interventions were implemented in 30 selected shehias (smallest administrative areas) in Pemba and Unguja, respectively, as part of a cluster randomized trial supported by the Schistosomiasis Consortium for Operational Research and Evaluation (SCORE) [17]. The impact of the interventions was monitored in annual cross-sectional surveys in 90 study schools and shehias from 2012 until 2017. The interventions reduced the *S*. *haematobium* prevalence in Zanzibar from 3.9% to 1.5% in 20–55 year old adults, and from 6.6% to 1.9% in 9–12 year old schoolchildren from 2012 to 2017. In 2017, heavy *S*. *haematobium* infection intensities (≥50 eggs per 10 ml urine) occurred in 0.1% of 20–55 year old adults and in 0.5% of 9- to 12-year old schoolchildren [19]. Hence, within the ZEST project, urogenital schistosomiasis was eliminated as public health problem from most schools and communities in Zanzibar in 2017 and the *S*. *haematobium* prevalence was significantly reduced [6,19]. While the SCORE trial was concluded in early 2017, MDA in schools and shehia communities continued under the lead of the Zanzibar NTD Program, and was implemented annually in 2017 and 2018. No MDA was conducted in 2019 due to procurement issues.

We aimed to monitor the *S*. *haematobium* prevalence in children and adults in Zanzibar from 2012 till 2020 and to assess recrudescence patterns and risk factors for infection across all age groups with a particular focus on the year 2020, after the 16-month treatment gap.

## Methods

### Ethics statement

The SCORE cluster randomized intervention trial, including annual cross-sectional surveys to assess the *S*. *haematobium* prevalence in 90 study schools and shehias in Pemba and Unguja from 2012 till 2018 was approved by the Zanzibar Medical Research Ethics Committee in Zanzibar, United Republic of Tanzania (ZAMREC, reference no. ZAMREC 0003/Sept/011), the Ethikkommission beider Basel (EKBB) in Basel, Switzerland (reference no. 236/22) and the Institutional Review Board of the University of Georgia in Athens, Georgia, United States of America (project no. 2012-10138-0). The trial is registered in the International Standard Randomized Controlled Trial Number register (ISRCTN; ISRCTN48837681). The cross-sectional surveys conducted in 2019 and 2020 obtained ethical approval from the Zanzibar Health Research Institute in Zanzibar (ZAHRI), United Republic of Tanzania (reference no. NO. ZAHREC/02/June/2019/36) and the Ethics Committee Northwest and Central Switzerland (EKNZ) in Basel, Switzerland (Project ID: Req-2019-00524). The surveys are registered in ISRCTN (ISRCTN17656730). All individuals participating in the cross-sectional surveys from 2011/12 until 2020 were informed in detail about the objectives and procedures of the studies and provided written informed consent for their participation. In the case of children below the age of 18, written consent was obtained from their parents or guardians.

### Study site

The Zanzibar islands (Pemba and Unguja) are a semi-autonomous part of the United Republic of Tanzania. Both islands are located in the Indian Ocean, about 30 km off the coast from the east African mainland [20]. The islands are divided into 11 districts, which are again subdivided into 388 small administrative areas, called shehias [21]. The estimated population of Zanzibar for 2019 was 1.6 million with a population density of 669 person/km^2^ in Unguja and 503 person/km^2^ in Pemba. The proportion of the population aged 0–17 years was 49.0% [21]. Public primary schools in Zanzibar contain the grades 1–6 and some schools additionally include a nursery school. The net enrolment rate in primary schools was 83.2% in 2014/15 and the percentage of people being employed or full-time student was 67.8% [22]. Among the total population of Zanzibar, 28.9% had access to an improved water supply source in 2014/15 and 59.0% had access to an own improved toilet facility in 2015/16 [22,23].

### Interventions for schistosomiasis elimination in Zanzibar

From 2012 to 2016, MDA was conducted biannually in Pemba and Unguja by the NTD Program of the Zanzibar Ministry of Health within the ZEST project [17]. The inhabitants of all shehias, excluding those located in the South district and in parts of the Urban district in Unguja, were offered praziquantel via community-wide treatment (CWT) or school-based treatment (SBT) approaches. During CWT, trained community drug distributors offered praziquantel (40 mg/kg) in a door-to-door approach to all community members aged >3 years that did not receive praziquantel in the same treatment round via SBT, or were not pregnant or severely sick, using a dose pole [24,25]. CWT was conducted biannually (twice a year) from April 2012 to November 2016 on both islands [6]. In SBT, trained teachers provided directly-observed praziquantel treatment to the children attending school on the day of treatment using a dose pole [24,25]. SBT was implemented biannually, from November 2013 until November 2016, with exception of 2014 where SBT was conducted only once a year in Pemba, but not at all in Unguja.

In addition to biannual MDA, 15 randomized shehias in Pemba and Unguja, respectively, received snail control interventions and 15 randomized shehias on each island received behaviour change interventions as part of the SCORE cluster randomized trial from 2012 till 2016. These interventions are described elsewhere in detail [6,17,19].

Once the SCORE trial and respective interventions were concluded in early 2017, the Zanzibar NTD Program continued the implementation of MDA. In 2017 and 2018, both CWT and SBT were conducted once a year. In 2019, no MDA was implemented, due to difficulties in procurement.

### Study design and participants

The SCORE study implemented from 2011/12 until 2017 was designed as a 5-year cluster randomized trial. The impact of biannual MDA alone or in combination with snail control or behavior change interventions was monitored annually with cross-sectional surveys in a total of 90 shehias and 90 schools in Pemba and Unguja, respectively [17]. Details of the design of the SCORE study, including the justification of the number of participants, are described elsewhere [17,19]. In brief, the SCORE study population included annually ~50 adults aged 20- to 55 years in each of the 90 shehias that were enrolled in parasitological community-based surveys (CBS) that also included a questionnaire component. Moreover, ~100 schoolchildren aged 9- to 12-years from grade 3 and 4 in each of the 90 study schools were enrolled annually in parasitological school-based surveys (SBS) that also included a questioning component. Additionally, in the years 2012 and 2017, ~100 schoolchildren from grade 1 in each of the 90 study schools were enrolled in the SBS [19].

After the SCORE study was concluded in early 2017, the annual cross-sectional surveys continued with the same design and sample sizes for children aged 9- to 12-years in the SBS and for adults aged 20- to 55-years in the CBS in 2018, 2019, and 2020 to monitor the impact of annual MDA in 2017 and 2018 and the treatment gap in 2019 on *S*. *haematobium* infections.

In 2018, only a SBS including schoolchildren aged 9- to 12-years from grade 3 and 4 as well as children from grade 1 was conducted, but no CBS.

In 2019 and 2020, we were interested not only in the age groups targeted by SCORE, but aimed to investigate the extent of *S*. *haematobium* infections in a broader age group. Hence, in the CBSs in the years 2019 and 2020, not only 50 individuals aged 20–55 years as in SCORE, but 70 individuals aged 15–100 years were sampled per shehia. Moreover, in the SBS, the original sample size of ~100 children from grades 3 and 4 in each study school was kept as in SCORE, and additionally 20 children each from nursery, grade 1, 2, 5, and 6 were added.

**Fig 1:**
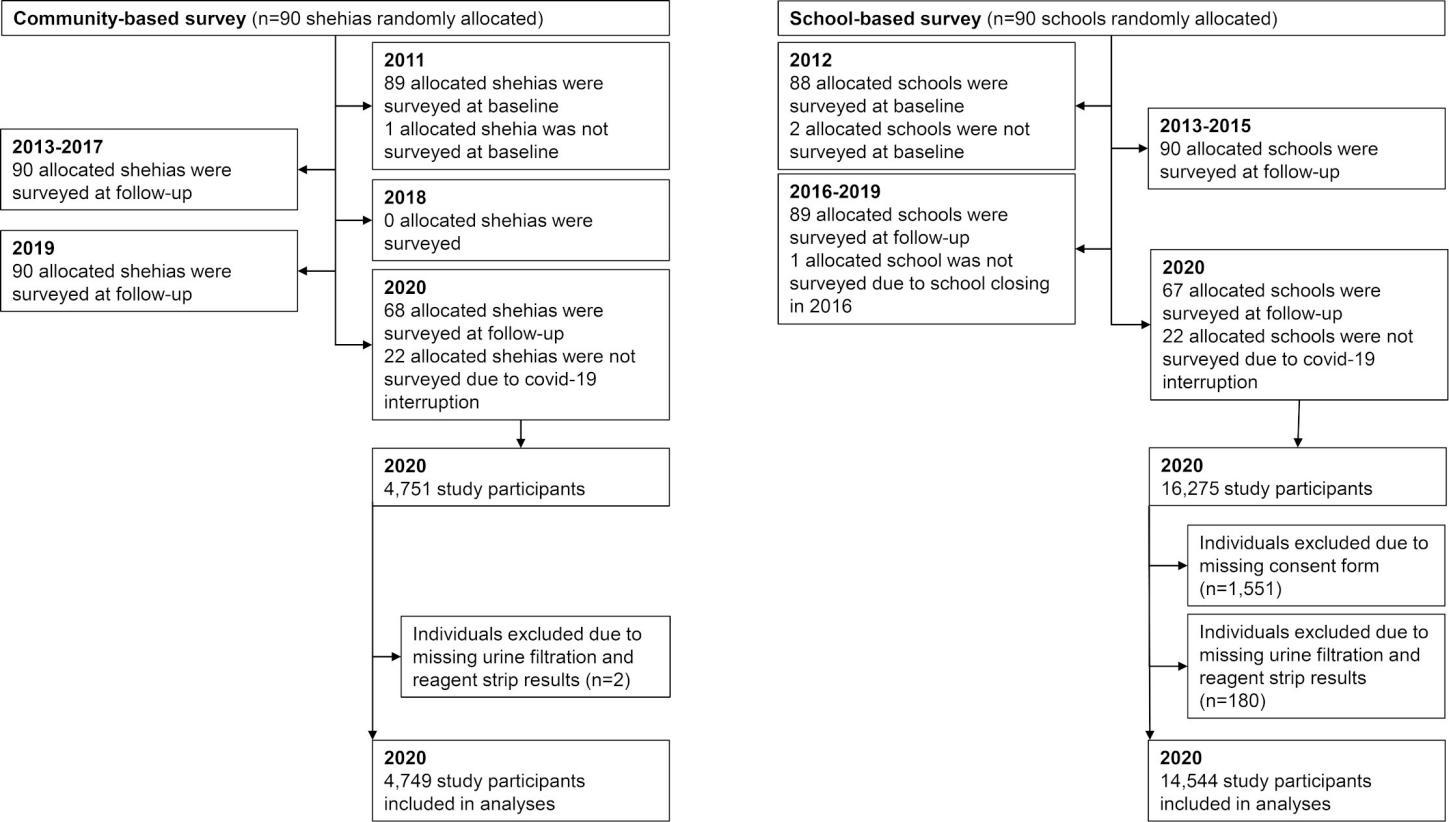
Flowchart of study design.

The baseline CBS was conducted in November and December 2011. The baseline SBS was conducted from February till April 2012. All other CBS and SBS were conducted between January and May every year. Unfortunately, in 2020, the CBS and SBS were not completed due to the Covid-19 pandemic. Therefore, in 2020, the surveys were limited to 68 shehias and 67 corresponding schools (Fig 1).

### Field procedures

Each year before the surveys commenced, the community leader (sheha) and head teacher of each study shehia and school, respectively, were invited to a meeting, where the procedures and aims of the forthcoming survey were explained and results from the previous survey were presented. The meeting participants were requested to inform their shehia population and school, respectively, about the purpose of the survey.

In the CBS, the survey teams visited each shehia for one day. In the years 2011–2017, data of ~50 adults per shehia were collected. In the years 2019 and 2020, data of ~70 adolescents and adults (aged 15–100 years) per shehia were collected. The randomization and selection of households and participants is described elsewhere in detail [17]. In all years, the study procedure was explained in Kiswahili to the members of the selected households by a trained fieldworker and the selected participants were asked to provide written consent by signing the informed consent form with their signature or thumbprint. Subsequently, they were invited to produce and submit a fresh urine sample and to answer questions from a pre-tested questionnaire. Besides the collection of demographic data, participants were asked about behaviors that might put them at risk of *S*. *haematobium* infection, for example their participation in the last MDA round, contact with natural freshwater, access to clean water, and travel history.

In the SBSs, each school was visited by the study team for two subsequent working days. The randomization of schoolchildren for participation is explained elsewhere in detail [17]. In brief, in each grade eligible for participation, girls and boys lined up stratified by sex, and every third child was selected until the requested number was reached. In 2012–2020, ~120 children from grades 3 and 4 were selected. In addition, in 2012, 2017 and 2018, ~120 children from grade 1 were selected. Finally, in 2019 and 2020, ~25 children each from grades 1, 2, 5 and 6 as well as from nursery were selected. The additional grades were included in 2019 and 2020 to obtain a better picture on the age profile of *S*. *haematobium* infections. The study procedure was explained in lay terms in Kiswahili to the enrolled children. Upon registration, the children were asked about their demographic data, their travel history, and in years with preceding MDA also about their participation in the last SBT round. All enrolled children received an information- and a consent-sheet for their parents or legal guardians to read and sign. On the next day, all children that submitted an informed consent form signed by their parents or legal guardians were given a plastic container (100 ml) to provide their fresh own urine sample.

All urine samples collected in CBS and SBS were produced between 10:00 and 14:00. The samples from Unguja were transferred to the laboratory of the NTD Program and all urine samples collected in Pemba were taken to the Public Health Laboratory–Ivo de Carneri (PHL-IdC).

### Laboratory procedures

The urine samples were examined in the laboratories on the day of collection. First, the samples were investigated for visible macrohematuria using a color chart and for microhematuria using reagent strips (Siemens Health Care AG, Zürich, Switzerland). Subsequently, each urine sample with a volume of at least 10 ml was vigorously shaken and filtered through a polycarbonate filter (Sefar Ltd., Bury, United Kingdom), which was then examined under a microscope for the presence of *S*. *haematobium* eggs by a well-experienced laboratory technician. The grade of macrohematuria and microhematuria, respectively, as well as the number of *S*. *haematobium* eggs for each participant was recorded on a laboratory form.

### Outcomes

The primary outcome variable was the *S*. *haematobium* infection status in study participants from Pemba and Unguja in each cross-sectional survey.

The secondary analyses covered the annual *S*. *haematobium* prevalence and infection intensity as well as microhematuria levels among 20-to 55-year old adults and 9- to 12-year old children in the years 2011/12 to 2020 at aggregated and shehia/school level. Moreover, we assessed the age-prevalence distribution of *S*. *haematobium* infections in the years 2019 and 2020, stratified by sex, and including study participants of all ages. Finally, we determined the odds of infection in relation to demographic and behavioral factors in 2020.

### Data management and statistical analyses

In all cross-sectional surveys from 2011/12 till 2020, data collected during registration in the SBS and laboratory examinations of the CBS and SBS were recorded on paper forms by the field enumerators and laboratory technicians, respectively, and subsequently double entered in electronic spreadsheets (Excel 2010, Microsoft) by data entry clerks in Zanzibar. In the years 2011/12 till 2019, paper questionnaire data collected in the CBS were entered into EpiInfo version 3.5.4 (Centers for Disease Control and Prevention, Atlanta, United States of America) by local staff in Zanzibar. In 2020, CBS questionnaire data were collected with the software Open Data Kit (https://opendatakit.org/) installed on Samsung Galaxy 4 tablets (Samsung Electronics, Seoul, South Korea). All data were cleaned and analyzed with StataIC 16 (StataCorp., Texas, United States of America) and R version 4.0.3 (www.rpoject.org).The data that support the findings of this study from 2011/12-2017 are openly available in ClinEpiDB. The dataset “Study: SCORE Zanzibar *S*. *haematobium* Cluster Randomized Trial” can be found at https://clinepidb.org/ce/app/record/dataset/DS_eddb4757ba. The data that support the findings of this study from 2018–2020 are within the manuscript and its supporting information (S1 Data and S1 Dictionary).

All study participants with a written informed consent and results of the urine filtration and/or reagent strip examination were included in the statistical analyses. A participant was considered *S*. *haematobium* positive if at least one egg per 10 ml urine was detected by the urine filtration method. Infections were stratified into light intensity (1–49 eggs per 10 ml urine) and heavy intensity (≥50 eggs per 10 ml urine) infections, according to WHO guidelines [26]. In the absence of a urine filtration result, a participant was considered *S*. *haematobium*-positive if the urine was microhematuria-positive, as detected by reagent strips. The intensity of microhematuria was graded into negative, trace, 1, 2 or 3, in line with the color code provided by the reagent strip`s manufacturer.

For the determination of the annual *S*. *haematobium* prevalence and infection intensity at aggregated and cluster level, only data from the 20-to 55-year old adults and from the 9-to 12-year old schoolchildren collected in the years 2011/12 to 2020 were included in the analyses. These restrictions were applied across the years, since during the SCORE study from 2011/12-2017, only data from these age groups were collected and we aimed to keep the annual data comparable.

For the differentiation of hotspot *versus* low prevalence areas, we defined a hotspot area as a shehia or school, which had a point prevalence of ≥10% in at least one of the study years and a low prevalence area as a shehia or school with point prevalences <10% throughout all study years from 2011/12 till 2020. Maps containing information about the spatial distribution of prevalence were created with R version 4.0.3. Coordinates of schools were collected with a handheld Garmin GPSMAP 62sc device (Garmin, Kansas City, USA). Shape files of shehias were provided by the Zanzibar Health Management Information System to the NTD Program of the Zanzibar Ministry of Health.

For the determination of the age profile of *S*. *haematobium* infections, participants of all ages of the 2019 and 2020 surveys were included in the analyses. Generalized estimating equation (GEE) models with exchangeable correlation structure were applied to estimate the odds ratios (OR) for *S*. *haematobium* infection. Separate models were run for the CBS and SBS, respectively, in 2020. The following “risk factors” were investigated as explanatory variables: sex (binary variable), age (categorical variable), travel history (binary variable), place of birth (categorical variable), living close to a natural freshwater body (binary variable; in CBS only), using water from natural freshwater bodies (binary variable; in CBS only), having a tap in or near the house (binary variable; in CBS only), having a well in or near the house (binary variable; in CBS only), and school grade (categorical variable; in SBS only). Besides the variables “place of birth” (excluded in both surveys due to collinearity), “use of water from natural freshwater bodies” (excluded in CBS due to collinearity), and “age” (excluded in SBS due to collinearity), all listed variables were included in the multivariable GEE models. In the models, 95% confidence intervals (CI) were used, and GEE with robust standard errors to account for clustering. Stratified by CBS and SBS, either the shehias (CBS) or the schools (SBS) were included in the model as clusters.

## Results

### Study flow and participant characteristics

The baseline CBS in 2011 was conducted in 89 among 90 allocated shehias in Pemba and Unguja; one allocated shehia was mistakenly not surveyed. In the CBSs implemented from 2013 to 2017 and in 2019, all 90 allocated shehias were surveyed. In 2020, the CBS was interrupted due to the Covid-19 pandemic, and only 68 among 90 allocated shehias were surveyed (Fig 1). In each of the CBS implemented from 2011 to 2017, around 4,400 adults aged 20–55 years participated. In the CBSs in 2019 and 2020, 6,300 and 4,749 individuals aged 15 years and older were enrolled.

The baseline SBS in 2012 was carried out in 88 among 90 allocated schools in Pemba and Unguja; two allocated schools were mistakenly not surveyed. In the SBSs implemented from 2013 to 2015, all 90 allocated schools were surveyed. Due to a school closing in 2016, the SBSs in 2016 till 2019 were conducted in 89 schools. Due to the Covid-19 pandemic and the associated interruption of survey activities in Zanzibar in March 2020, the SBS in 2020 was limited to 67 schools (Fig 1). In the SBSs conducted from 2012 to 2018, around 9,700 schoolchildren aged 9–12 years participated each year. In 2019 and 2020, a total of 19,559 and 14,544 children, respectively, were enrolled. Characteristics of the study participants from each CBS and SBS, respectively, are presented in Table 1.

**Table 1:** Characteristics of the study participants from community-based survey (CBS) and school-based survey (SBS) by year.

Survey	Characteristic	Stratification	2011/12	2013	2014	2015	2016	2017	2018	2019	2020
**CBS**	**Shehias - n**		89	90	90	90	90	90	0	90	68
** **	**Participants with outcome data - n**		3974	4378	4462	4469	4474	4487	0	6300	4749
** **	**Sex - n (%)**	Female	2835 (71)	2671 (61)	2821 (63)	2478 (55)	2395 (54)	2133 (48)	0	3327 (53)	3010 (63)
** **	** **	Male	1136 (29)	1707 (39)	1641 (37)	1991 (45)	2079 (46)	2354 (52)	0	2973 (47)	1739 (37)
** **	**Age group - n**	≤19 year old adults	0	0	0	0	0	0	0	1205	627
** **	** **	20-55 year old adults	3974	4378	4462	4469	4474	4487	0	4742	3717
** **	** **	≥56 year old adults	0	0	0	0	0	0	0	344	372
**SBS**	**Schools - n**		88	90	90	90	89	89	89	89	67
** **	**Participants with outcome data - n**		8931	9379	9595	9803	9725	10632	9662	19559	14544
** **	**Sex - n (%)**	Female	4664 (52)	5041 (54)	5098 (53)	5093 (52)	5122 (53)	5308 (50)	4903 (51)	9814 (50)	7407 (51)
** **	** **	Male	4267 (48)	4338 (46)	4497 (47)	4710 (48)	4603 (47)	5324 (50)	4759 (49)	9745 (50)	7137 (49)
** **	**Age group - n**	≤8 year old children	0	0	0	0	0	0	0	4622	3606
		9-12 year old children	8931	9379	9595	9803	9725	10632	9662	12017	9081
		≥13 year old children	0	0	0	0	0	0	0	2906	1856

### Change in annual *S*. *haematobium* prevalence and intensity and microhematuria levels

**Fig 2:**
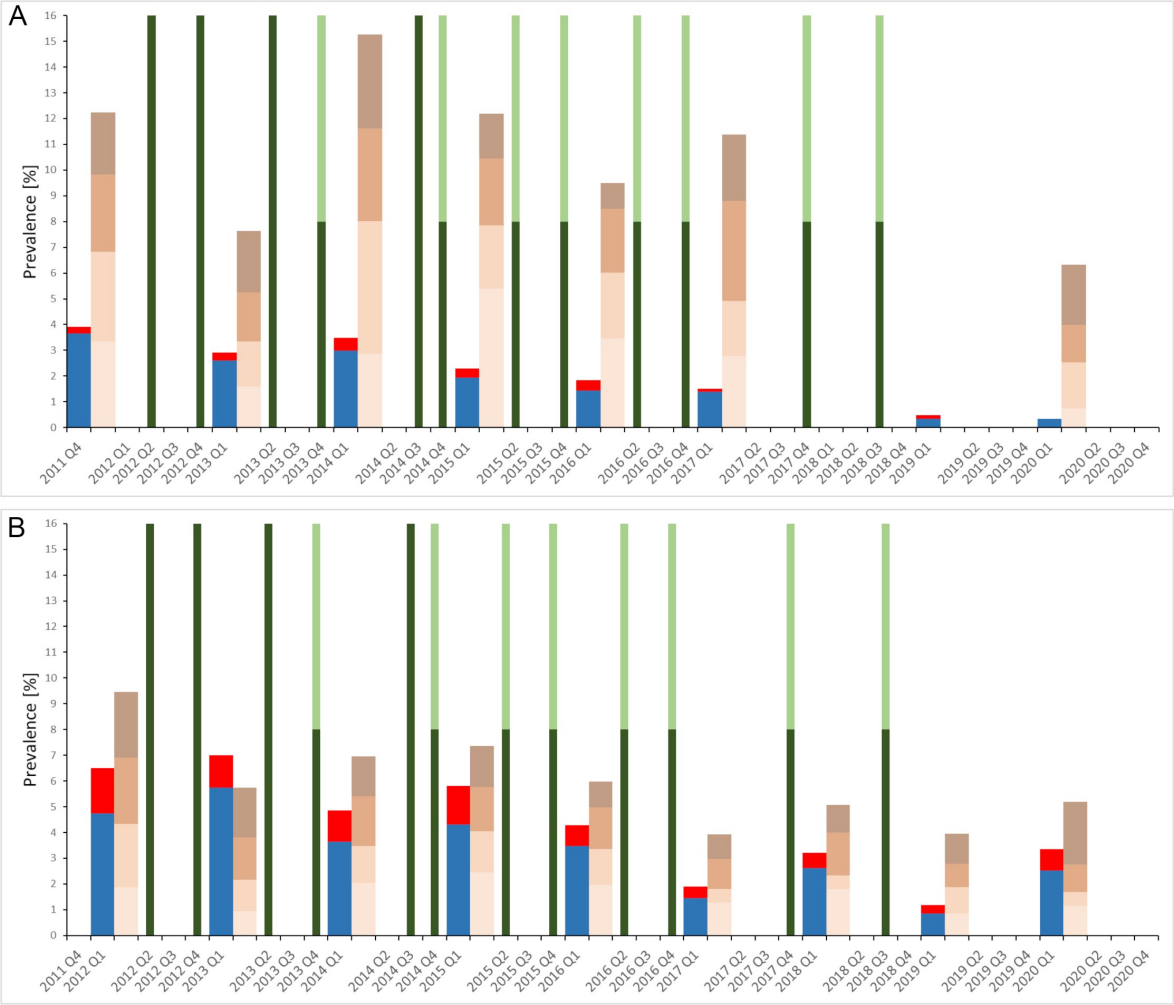
Prevalence and intensity of *S*. *haematobium* infections, microhematuria levels, and implementation of MDAs in Zanzibar from 2011/12 until 2020. The data show the *S*. *haematobium* prevalence and infection intensity, the levels of microhematuria, and implementation of MDA per annual quartile (Q). A: 20- to 55-year old adults; B: 9- to 12-year old schoolchildren. Blue: light intensity infection prevalence; red: heavy intensity infection prevalence. Very light brown: microhematuria graded trace; light brown: microhematuria graded 1; brown: microhematuria graded 2; dark brown: microhematuria graded 3. Dark green: community-wide treatment (CWT) only; dark and light green: community-wide (CWT) and school-based treatment (SBT).

Fig 2 shows that in the CBSs implemented from 2011 till 2020, the overall *S*. *haematobium* prevalence decreased constantly among the 20–55 year old adults from 3.9% in 2011 to 0.4% in 2020. An exception was 2014, when the prevalence increased by 0.5%-points (from 3.0% to 3.5%) compared with the previous year. The relative difference in the prevalence from 2011 to 2020 was -91%. Heavy intensity infections were found in 0.3% of participants at baseline in 2011, reached a maximum of 0.5% in 2014, and declined to zero in 2020.

Microhematuria levels in 20–55 year old adults were higher than the *S*. *haematobium* prevalence in all study years. From 2011 till 2020, the overall microhematuria among the 20–55 year old adults decreased from 12.2% in 2011 to 6.3% in 2020. A higher overall microhematuria level than in 2011 was observed in 2014 (15.3%). The highest grading of microhematuria (3) ranged between 1.0% and 3.7% and no decreasing trend in heavy microhematuria was observed over the study period.

In the SBS, a decreasing trend of the overall *S*. *haematobium* prevalence among the 9–12 year old children was observed from 2012 (6.6%) to 2019 (1.2%), with some inconsistent upturns in certain years (Fig 2). The relative difference in the prevalence from 2012 to 2019 was -82%. At baseline in 2012, heavy intensity infections were detected in 1.8% of participants and decreased to 0.3% in 2019 (-83%). A prominent rebound in the overall *S*. *haematobium* infections and intensity was observed after the 16-month treatment gap in 2020, when the prevalence of infections rose back from 1.2% to 3.4% (relative difference +183%) and heavy intensity infections rebounded from 0.3% to 0.8% (relative difference +167%).

Microhematuria levels in 9–12 year old schoolchildren were higher than the *S*. *haematobium* prevalence in all study years, except in 2013. In line with the *S*. *haematobium* prevalence, a decreasing trend of the overall microhematuria levels was observed from 2012 (9.5%) till 2019 (4.0%). The relative difference in the percentage of the overall microhematuria from 2012 to 2019 was -58%. At baseline in 2012, heavy microhematuria levels (3) were detected in 2.6% of participants and decreased to 1.2% in 2019 (-54%). A rebound in the overall microhematuria level was observed after the 16-month treatment gap in 2020, when the overall levels rose back from 4.0% to 5.2% (relative difference +30%) and heavy microhematuria rebounded from 1.2% to 2.5% (relative difference +108%).

### Spatial heterogeneity of *S*. *haematobium* prevalence

**Fig 3:**
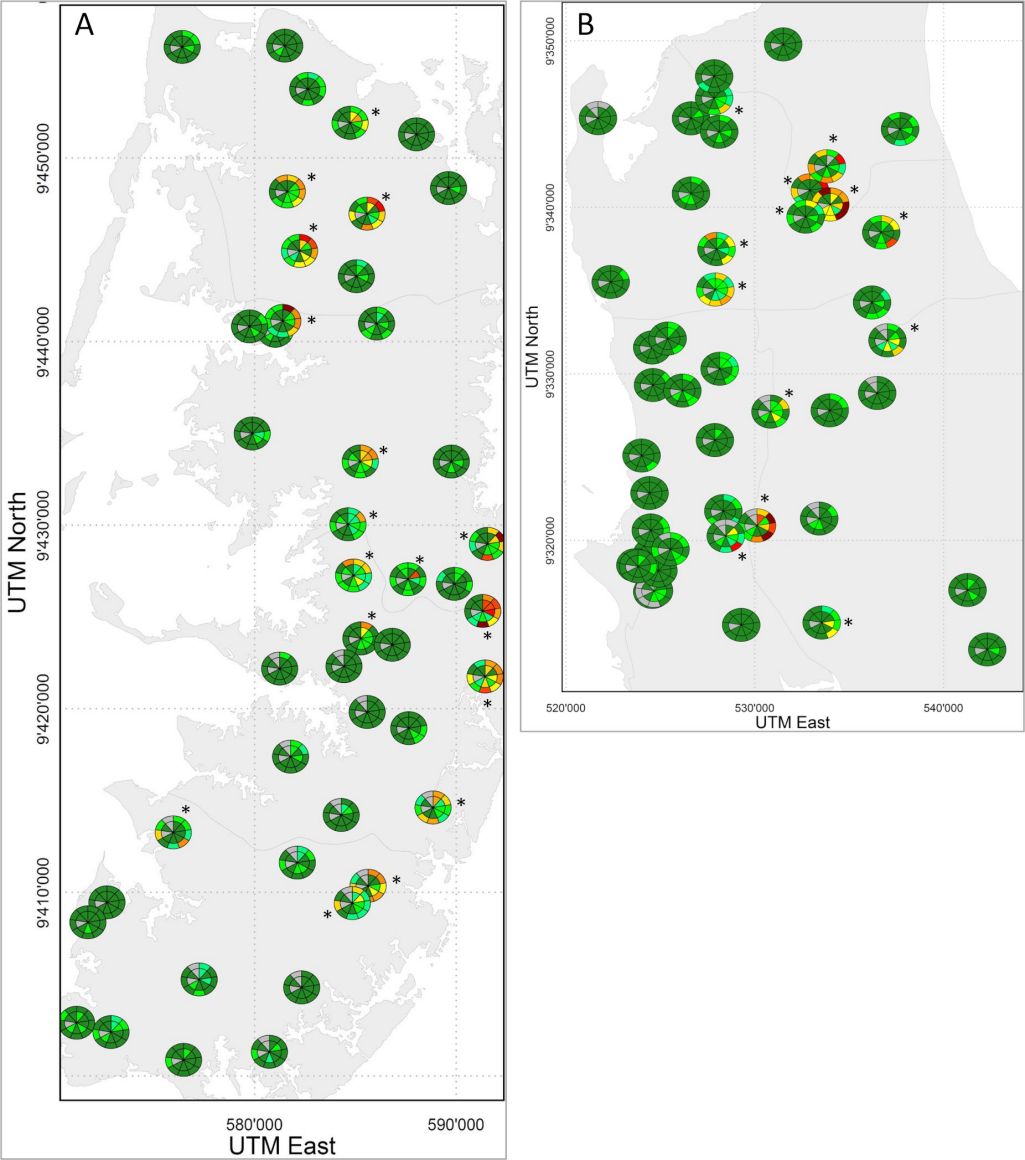
**Maps indicating prevalence of *S*. *haematobium* infections from 2011/12 till 2020 in spatial clusters in Pemba (A) and Unguja (B).** Data are *S*. *haematobium* prevalence in 45 schools/shehias (clusters) per island, stratified by population group and island. A: Pemba; B: Unguja. Data are presented per year starting from 2011/12 (12 o`clock position) to 2020. Inner circle: 20- to 55-year old adults; outer circle: 9- to 12-year old schoolchildren. The colors show the prevalence from dark green as lowest prevalence to dark red as highest prevalence, and grey indicates missing. The * represents a hotspot school and/or community. Maps containing information about the spatial distribution of prevalence were created with R version 4.0.3. Coordinates of schools were collected with a handheld Garmin GPSMAP 62sc device (Garmin, Kansas City, USA). Shape files of shehias were provided by the Zanzibar Health Management Information System to the Neglected Diseases Program of the Zanzibar Ministry of Health.

**Fig 4:**
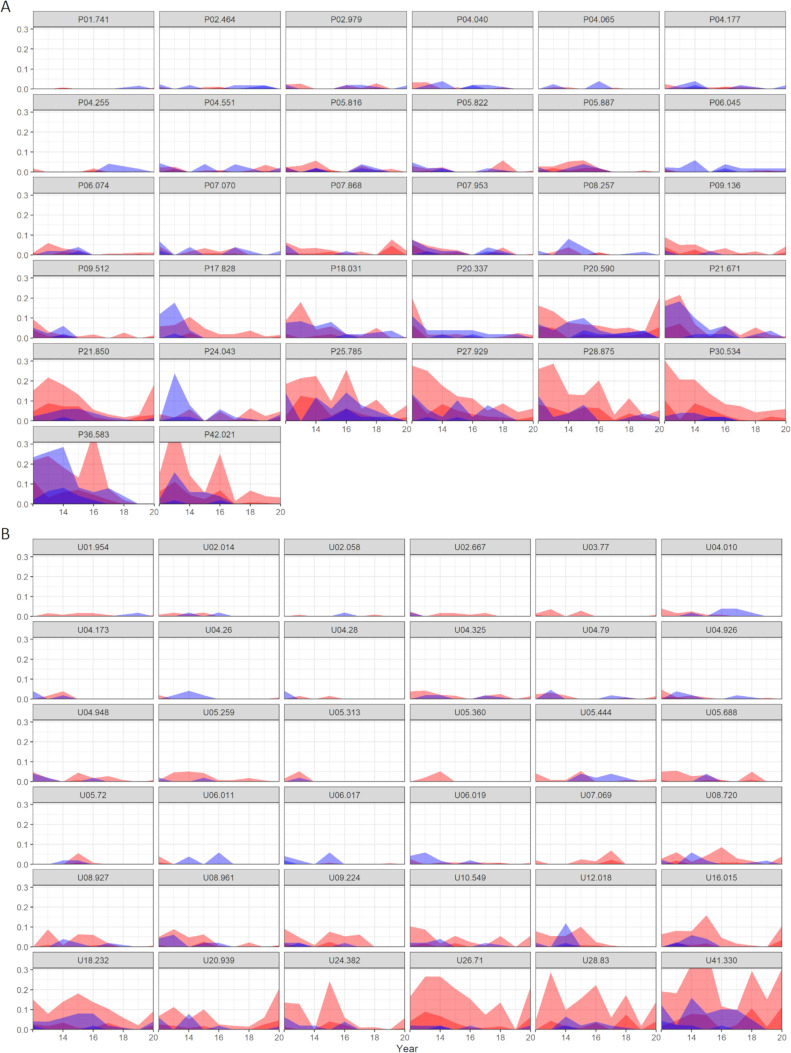
**Prevalence of *S*. *haematobium* infections stratified by infection intensity levels in Zanzibar from 2011/12 until 2020 per spatial cluster in Pemba (A) and Unguja (B).** Data are proportion of *S*. *haematobium* infection intensity in schoolchildren and adults in each surveyed cluster on each island. A: Shehias and schools in Pemba; B: Shehias and schools in Unguja. X-axis: year of survey (2011/12 to 2020); y-axis: proportion infection intensity. Dark blue: heavy intensity infection among 20- to 55-year old adults; light blue: light intensity infection among 20- to 55-year old adults. Dark red: heavy intensity infection among 9- to 12-year old schoolchildren; light red: light intensity infection among 9- to 12-year old schoolchildren.

The geographical distribution of the shehias and schools, and the *S*. *haematobium* prevalence in 20–55 year-old adults and 9–12 year-old children from 2011/12 to 2020 is indicated in Fig 3. A total of 71 shehias and 61 schools showed a prevalence <10% throughout all study years and were therefore defined as low prevalence areas, whereas 19 shehias and 29 schools crossed the 10% line in one or several years and were therefore considered hotspot areas. Fig 4 shows that the hotspot areas were more prone to recrudescence of *S*. *haematobium* infections and heavy intensity infections in one year or the other, and mainly responsible for the strong rebound observed after the 16-month treatment gap in 2020. While the rebound of the overall *S*. *haematobium* prevalence in schools from 2019 to 2020 was +183% (from 1.2% to 3.4%), the rebound in hotspot areas was +225% (from 2.8% to 9.1%) and the rebound in the low prevalence areas was +75% (from 0.4% to 0.7%).

### Age prevalence distribution

**Fig 5:**
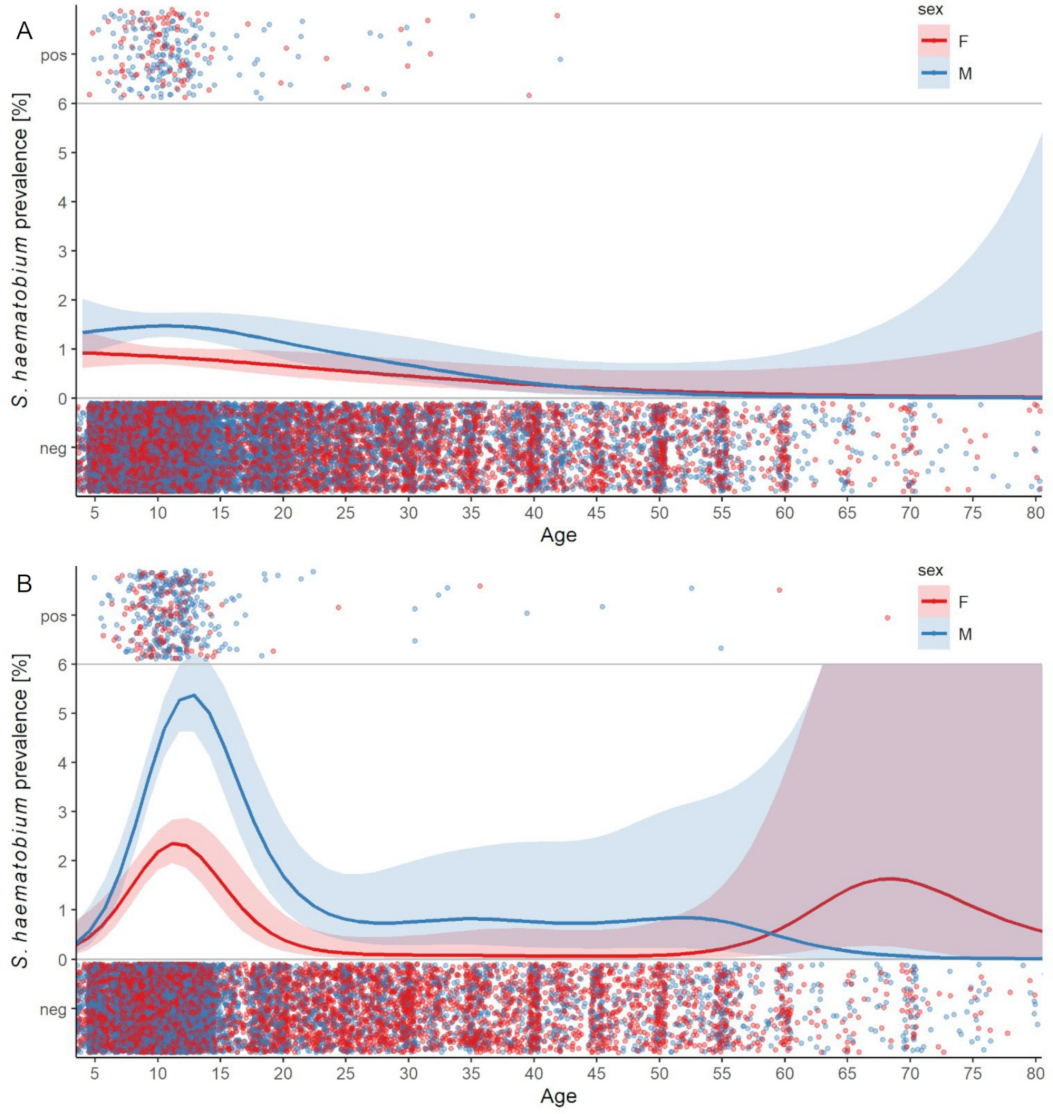
***S*. *haematobium* prevalence across all ages, stratified by sex, after 11 rounds of MDA in 2019 (A) and a 16-month treatment gap in 2020 (B).** The blue (male) and red (female) dots in the upper part (pos) of each figure, each represent a *S*. *haematobium*-positive urine sample from an individual of a certain age (x-axis). The blue (male) and red (female) dots in the lower part (neg) of each figure, each represent a *S*. *haematobium*-negative urine sample prom an individual of a certain age (x-axis). The blue (male) and red (female) lines in the middle part of each figure represent the predicted *S*. *haematobium* age-prevalence. The shading around the lines represent 95% confidence bands.

Fig 5 shows the age prevalence distribution of *S*. *haematobium* infections stratified by sex and in relation to the sampling effort in 2019 (Fig 5A) and 2020 (Fig 5B). In 2019, the *S*. *haematobium* infection levels were below 2.0% in both, male and female students, and community members. The prevalence was significantly higher in boys aged 8 to 16 years, compared with female students of the same age. No participant older than 42 years was tested *S*. *haematobium*-positive. In 2020, after the 16-month treatment gap, a prominent increase in prevalence was observed in 9–16 year-old schoolchildren of both sexes. Among the whole study population and all ages younger than 60 years, male participants had higher levels of infections than female participants did.

### Risk factors for *S*. *haematobium* infection in 2020

**Fig 6:**
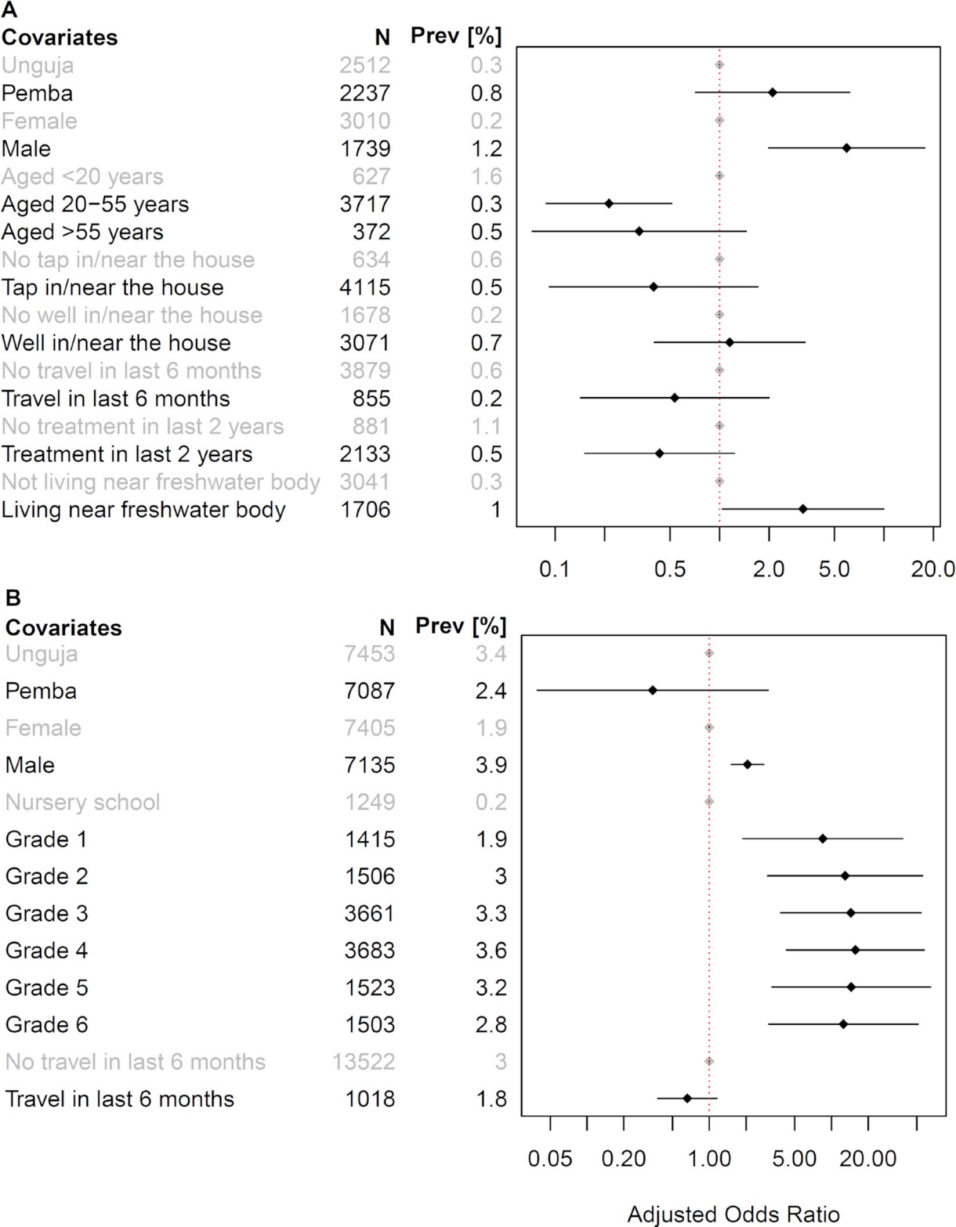
**Multivariable analysis of risk factors for *S*. *haematobium* infections in adults (A) and schoolchildren (B) in Zanzibar in 2020.** The figure shows the odds ratios for a *S*. *haematobium* infection adjusted for different risk factors. Grey dots indicate the reference categories. N: total number of participants analyzed per group; Prev [%]: *S*. *haematobium* prevalence per group.

In the CBS conducted in 2020, men had significantly higher chances of harboring a *S*. *haematobium* infection compared with women (OR: 6.24, 95% CI: 1.96–19.60, p = 0.002) (Fig 6A). Adults aged 20–55 years had significantly lower odds of having a *S*. *haematobium* infection than community members aged below 20 years (OR: 0.30, 95% CI: 0.13–0.69, p = 0.004). A *S*. *haematobium* infection was significantly associated with living in close proximity to a natural freshwater body (OR: 2.90, 95% CI: 1.12–7.54, p = 0.029). Having a tap in or near the house, travel outside the home island over the past 6 months, and praziquantel treatment within the last two years resulted in non-significant lower odds of *S*. *haematobium* infection.

In the SBS, boys had significantly higher chances of having a *S*. *haematobium* infection compared with girls (OR: 2.06, 95% CI: 1.52–2.78, p < 0.001) (Fig 6B). In each of the school grades 1 to 6, children had a higher chance of harboring a *S*. *haematobium* infection compared with children who attended nursery school, with children from grade 1 showing the lowest odds ratio (OR: 8.49, 95% CI: 1.89–38.16, p = 0.005) and children from grade 4 showing the highest odds ratio (OR: 15.70, 95% CI: 4.30–57.35, p < 0.001). A recent travel history outside the home island resulted in non-significantly lower odds of *S*. *haematobium* infection.

## Discussion

Considerable progress towards the elimination of urogenital schistosomiasis was made by the ZEST project from 2012 till 2017, when biannual praziquantel MDA alone or with additional snail control or behaviour change interventions were applied. Annual MDA was continued across Zanzibar in 2017 and 2018, but not in 2019. We monitored the *S*. *haematobium* prevalence and microhematuria levels from 2012 until 2020 and assessed recrudescence patterns and risk factors for infection across all age groups with a particular focus on the year 2020, after the 16-month treatment gap.

We found that the apparent overall *S*. *haematobium* prevalence among adults decreased from 3.9% at baseline in 2011 to 0.35% in 2020. Overall microhematuria levels in adults decreased from 12.2% in 2011 to 6.3% in 2020. Microhematuria levels in adults were remarkably higher than the *S*. *haematobium* prevalence measured by egg output by urine filtration microscopy and also than the microhematuria levels in children. This observation might indicate that either many adults suffer from chronic morbidity due to urogenital schistosomiasis that is not reflected by the number of eggs excreted in urine, or, microhematuria in adults might be caused by reasons unrelated to schistosomiasis such as urinary tract infections, bladder stones, sickle cell anemia, or pregnancy or menstrual bleeding in females [27–29].

The apparent overall *S*. *haematobium* prevalence among schoolchildren decreased from 6.6% at baseline in 2012 to 3.4% in 2020 and microhematuria levels from 9.5% to 5.2%, respectively. However, an even lower *S*. *haematobium* prevalence of 1.2% and microhematuria levels of 4.0% were observed in schoolchildren in 2019. In 2020, the considerable rebound in the overall prevalence and also infection intensity was caused by certain hotspot areas, while most areas had very low prevalences throughout the study period and also after the 16-month treatment gap. The hotspot areas showed an unstable and undulating *S*. *haematobium* prevalence pattern over the years from 2011/12 to 2020. Rapid reinfection after praziquantel treatment and the persistence of hotspots are well-known challenges for the sustainable control and elimination of schistosomiasis [4,30–33].

Most of the hotspot areas in Pemba and Unguja were not geographically isolated but neighbored with other hotspot areas. Clustering of *S*. *haematobium* infections in specific areas has also been observed in other studies, for example in Kenya [30,34]. The likelihood of reinfection is strongly dependent on the environment of regions and the force of transmission [9,35,36]. For example, an earlier study in some of the hotspot areas in Unguja conducted in 2014 showed that there were considerably more natural freshwater bodies in total and also more natural fresh water bodies containing the intermediate host snail transmitting *S*. *haematobium* compared with investigated low-prevalence areas [37].

In our study, the rebound in *S*. *haematobium* (re)infections from 2019 to 2020 was most prominent in children aged 8–16 year-old, and in boys. The latter observation is in line with another study from Senegal, which reports a higher *S*. *haematobium* reinfection rate in boys compared with girls after treatment with praziquantel [38]. However, the full age-profile of *Schistosoma* infections remains poorly researched, particularly in populations that received several rounds of MDA. We showed that in 2019, after 11 rounds of MDA in Zanzibar, infection levels across all age groups from four to 80 years were extremely low and a “real” age-prevalence distribution was inexistent. The “typical” age-prevalence curve, reported in reviews with a peak of *S*. *haematobium* infections occurring in children aged 8–15 years and a subsequent decrease and stabilization of a low prevalence in adult age [39,40], reformed quickly in the 16-month treatment gap in Zanzibar until early 2020.

Our risk factor analyses in 2020 indicated that men and boys had significantly higher odds of a *S*. *haematobium* infection than women and girls (OR: 6.24 and OR: 2.06, respectively). The observations that male individuals are more likely to harbor schistosomiasis is in line with previous surveys in Zanzibar and elsewhere [18,41–44]. Male individuals may be more engaged in behaviors that expose them to *S*. *haematobium* contaminated freshwater, such as swimming, playing and fishing [41,45,46]. In Pemba and Unguja, where Islam is the predominant religion, men also often use the rivers and ponds for ablution before their prayers. School aged children might get in frequent contact with contaminated freshwater when doing household chores such as washing dishes or clothes at the rivers and ponds near their home, or while engaging in leisure activities such as swimming and playing [47,48]. While boys might expose themselves more frequently to water than girls and thus acquire infections more often and quickly, we also observed that in line with their religious beliefs, girls frequently keep their clothes on while bathing or swimming, which might impose a certain barrier for cercarial penetration.

The risk factor analysis of adults in the CBS showed significantly elevated odds of *S*. *haematobium* infection for individuals who reported to live in close proximity to natural freshwater bodies (OR: 2.90). This observation confirms earlier reports from Zanzibar, where the proximity of households or schools to freshwater bodies containing the intermediate host snails was associated with *S*. *haematobium* infections [37,45]. Travel outside the home island, for example to the sister island, or mainland Tanzania, or other African countries, was not a significant risk factor in our study. Hence, at least to date, importation of urogenital schistosomiasis to Zanzibar does not seem to be a challenge and threat for elimination efforts. This finding is in contrast to reports from the neighboring island Mafia, where urogenital schistosomiasis is considered as an imported disease [49]. Also, access to clean water by having a tap or well near or at the home was not significantly associated with a *S*. *haematobium* infection in our study. This is in contrast to assumptions that access to clean water can improve the situation in endemic countries and reduce exposure to schistosomes [42,50]. However, one has to keep in mind that many drivers for infection are complex, interconnected and multidirectional and that a systems epidemiology approach is necessary to put them into context [51].

A limitation of our large-scale community- and school-based study that involved more than 10,000 people annually is the yet small number of individuals and particularly adults that were diagnosed *S*. *haematobium*-positive. While this is an expected impediment when working in an elimination setting, the low infection numbers rendered the risk factor assessment difficult and resulted in huge relative differences in the *S*. *haematobium* prevalence between some years. Moreover, we used basic parasitological methods (reagent strips and urine filtration microscopy) to detect *S*. *haematobium* infections, which are not very sensitively detecting very light intensity infections [52]. Hence, many more people than identified might have carried a very light intensity infection, thereby biasing associations and resulting in an underestimation of the true prevalence.

Zanzibar has made great achievements towards schistosomiasis elimination over the past years applying population-based interventions. The current evidence that the *S*. *haematobium* prevalence and morbidity rebound quickly over the period of a 16-month treatment gap in hotspot areas but remain low in areas with a weaker force of transmission, emphasizes the need to rethink carefully future elimination strategy approaches. Clearly, due to the spatial heterogeneity of *S*. *haematobium* infections in Zanzibar and the varying risk of reinfection, future interventions aiming for interruption of *S*. *haematobium* transmission need to consider the micro-epidemiology of the islands and be adapted to it [19]. We hence suggest that interventions targeting hotspot areas in Zanzibar should be re-intensified and ideally include biannual MDA, snail control, behavior change and, in a multi-sectoral approach, improved access to safe water and sanitation. In low prevalence areas, future efforts might shift from MDA towards surveillance-response, including risk-based test-and-treat approaches using new and sensitive point-of-care diagnostic tools, xenomonitoring, focal snail control and health communication to ensure the identification and treatment of those infected but to avoid overtreatment and treatment fatigue of the healthy population. Setting up an effective surveillance-response system in areas that are or become low prevalence areas, will also help to sustain the gains made by reacting to any outbreak and sign of recrudescence in time and thus to ultimately progress towards a sustained interruption of *S*. *haematobium* transmission across Zanzibar. Our results also indicate that interruption of MDA and potentially other interventions, for example caused by a lack of donor funding, procurement issues, or the current Covid-19 pandemic, can result in a rapid resurgence of transmission and disease that needs to be reacted to in time. Finally, our study highlights the need for more research on the sustainability of the gains of control and on the criteria and conditions required for stopping schistosomiasis MDA.

## Supporting information

S1 Dataset Data that support the findings of this study from 2018–2020. (CSV)

S1 Dictionary Data dictionary that explains the data collected from 2018–2020. (TXT)
